# Correction: Understanding stage of innovation of invasive procedures and devices: protocol for a systematic review and thematic analysis

**DOI:** 10.1136/bmjopen-2021-057842corr1

**Published:** 2022-08-26

Scroggie DL, Elliott D, Cousins S, *et al*. Understanding stage of innovation of invasive procedures and devices: protocol for a systematic review and thematic analysis. *BMJ Open* 2022;12:e057842. doi: 10.1136/bmjopen-2021-057842

The funding information in the published article was incorrect. The complete funding details are as follows:

This study was supported by the Medical Research Council [MR/S001751/1] and NIHR Biomedical Research Centre at University Hospitals Bristol and Weston NHS Foundation Trust and the University of Bristol.

