# Supplementary material for: Understanding stage of innovation of invasive procedures and devices: protocol for a systematic review and thematic analysis
Source: BMJ Open. 2022 Feb 10;12(2):e057842. doi: 10.1136/bmjopen-2021-057842 (PMC8845321; doi:10.1136/bmjopen-2021-057842)
Supplement: Supplementary data [file bmjopen-2021-057842supp002.pdf]

Search Strategy for Ovid MEDLINE

	Searches
1	((stage or phase or framework) adj2 (IDEAL or development or innovat*)).ti,ab.
2	IDEAL-D.ti,ab,kf,hw.
3	1 or 2
4	(invasive or incision or cut or percutaneous or puncture or (natural adj1 orifice)).ti,ab,kf,hw.
5	(endoscop* or colonoscop* or gastroscop* or thoracoscop* or laparoscop* or arthroscop* or bronchoscop*).ti,ab,kf,hw.
6	(catheter or scalpel or surgery or surgical or operat* or interventional).ti,ab,kf,hw.
7	(device* or implant* or prosthe* or robot*).ti,ab,kf,hw.
8	exp Specialties, Surgical/ or exp Endoscopy/ or Radiology, Interventional/ or exp Surgical Procedures, Operative/ or exp "prostheses and implants"/
9	4 or 5 or 6 or 7 or 8
10	(guideline* or guidance or recommendation* or proposal* or regulation* or advice or framework or typology or model or algorithm or evaluat* or determin* or practical method* or method* or concept* or hinder* or hindrance* or difficult* or problem*).ti,ab.
11	exp Guidelines as Topic/ or exp Classification/
12	10 or 11
13	3 and 9 and 12
14	limit 13 to english language
