# Supplementary material for: Understanding stage of innovation of invasive procedures and devices: protocol for a systematic review and thematic analysis
Source: BMJ Open. 2022 Feb 10;12(2):e057842. doi: 10.1136/bmjopen-2021-057842 (PMC8845321; doi:10.1136/bmjopen-2021-057842)
Supplement: Author's manuscript [file bmjopen-2021-057842.draft_revisions.pdf]

BMJ Open

BMJ Open is committed to open peer review. As part of this commitment we make the peer review history of every article we publish publicly available.

When an article is published we post the peer reviewers' comments and the authors' responses online. We also post the versions of the paper that were used during peer review. These are the versions that the peer review comments apply to.

The versions of the paper that follow are the versions that were submitted during the peer review process. They are not the versions of record or the final published versions. They should not be cited or distributed as the published version of this manuscript.

BMJ Open is an open access journal and the full, final, typeset and author-corrected version of record of the manuscript is available on our site with no access controls, subscription charges or pay-per-view fees (http://bmjopen.bmj.com).

If you have any questions on BMJ Open's open peer review process please email info.bmjopen@bmj.com

BMJ Open

Understanding stage of innovation of invasive procedures and devices: protocol for a systematic review and thematic analysis

Journal:	*BMJ Open*
Manuscript ID	bmjopen-2021-057842
Article Type:	Protocol
Date Submitted by the Author:	29-Sep-2021
Complete List of Authors:	Scroggie, Darren; NIHR Bristol Biomedical Research Centre; University Hospitals Bristol and Weston NHS Foundation Trust<br>Elliott, Daisy; NIHR Bristol Biomedical Research Centre<br>Cousins, Sian; NIHR Bristol Biomedical Research Centre<br>Avery, Kerry; NIHR Bristol Biomedical Research Centre<br>Blazeby, Jane; NIHR Bristol Biomedical Research Centre; University Hospitals Bristol and Weston NHS Foundation Trust<br>Blencowe, Natalie; NIHR Bristol Biomedical Research Centre; University Hospitals Bristol and Weston NHS Foundation Trust
Keywords:	QUALITATIVE RESEARCH, Clinical governance < HEALTH SERVICES ADMINISTRATION & MANAGEMENT, STATISTICS & RESEARCH METHODS, SURGERY, THERAPEUTICS

SCHOLARONE™
Manuscripts

Understanding stage of innovation of invasive procedures and devices: protocol for a systematic review and thematic analysis

Darren L Scroggie [a,b] darren.scroggie@bristol.ac.uk
Daisy Elliott [a] daisy.elliott@bristol.ac.uk
Sian Cousins [a] sian.cousins@bristol.ac.uk
Kerry NL Avery [a] kerry.avery@bristol.ac.uk
Jane Blazeby [a,b] j.m.blazeby@bristol.ac.uk
Natalie S Blencowe [a,b] natalie.blencowe@bristol.ac.uk

Corresponding author:
Darren L Scroggie
Bristol Centre for Surgical Research
Bristol Medical School
Department of Population Health Sciences
Canynge Hall
39 Whatley Road
Bristol
BS8 2PS
United Kingdom
darren.scroggie@bristol.ac.uk

Guarantor:
Natalie S Blencowe
Bristol Centre for Surgical Research
Bristol Medical School
Department of Population Health Sciences
Canynge Hall
39 Whatley Road
Bristol
BS8 2PS
United Kingdom
natalie.blencowe@bristol.ac.uk

[a] National Institute for Health Research
Bristol and Weston Biomedical Research Centre
Bristol Centre for Surgical Research
Bristol Medical School
Department of Population Health Sciences
Canynge Hall
39 Whatley Road
Bristol
BS8 2PS
United Kingdom

[b] University Hospitals Bristol and Weston NHS Foundation Trust
Bristol
BS2 8HW
United Kingdom

Word count: 1755

For peer review only

ABSTRACT

Introduction

Surgical innovation has generally occurred in an unstructured and unstandardised manner. This has led to unnecessary exposure of patients to harm, research waste, and an inadequate evidence base. To promote evaluation of surgical innovations, the IDEAL (Idea, Development, Exploration, Assessment, Long-term follow-up) Collaboration provided a structured set of recommendations based on the stage of innovation of a procedure or device. Despite further refinements and guidance, adoption of the IDEAL recommendations has been slow; an important reason may be that determining the stage of innovation of a novel procedure or device is often difficult. To facilitate evaluation of surgical innovations, there is a need for a detailed insight into what stage of innovation means, and how it can be determined. The aim of this study is to understand the concept of stage of innovation as reported in the literature.

Methods and analysis

A systematic review will be conducted. Ovid MEDLINE, Ovid Embase, CINAHL and INAHTA online bibliographic databases will be searched. A broad range of article types will be eligible, including reports of primary and secondary research, guidelines, and opinion pieces; conference abstracts and non-English language articles will be excluded. Other articles will be detected from citations within eligible articles and suggestions from experts in surgical innovation. Data will be extracted regarding approaches to evaluating surgical innovation, methods for determining stage of innovation, properties of innovations which could infer their stage of innovation, definitions of discrete stages, and potential sources of stage-related information. A thematic analysis will be conducted, and findings summarised in a narrative report.

For peer review only

Ethics and dissemination

Ethical approval will not be required. This systematic review will be published in a peer-reviewed journal and presented at appropriate conferences.

Registration

This systematic review is registered with PROSPERO (CRD42021270812).

For peer review only

ARTICLE SUMMARY

Strengths and limitations of this study

- A comprehensive search strategy and inclusive eligibility criteria will maximise the breadth of data obtained
- Thematic analysis is well-suited for searching for meaning and current understanding regarding abstract concepts such as stage of innovation
- An improved understanding of stage of innovation may support wider adoption of approaches to evaluating surgical innovations
- This study will be limited to articles written in the English language

For peer review only

INTRODUCTION

Progress in surgical practice has been driven by innovation. The development and implementation of new ideas has led to improvements of older procedures, as well as entirely novel approaches, such as the Ozaki procedure for aortic valve disease.[1] Innovation of surgical techniques has frequently been accompanied by innovation of related medical devices. For example, endoluminal surgical techniques and deployable artificial heart valves have resulted in effective and safe treatment options for otherwise untreatable patient cohorts.[2] Whilst innovation aims to improve outcomes, it is also inevitably associated with risks and potential hazards: the exposure of patients to unknown risks of harms has been an intrinsic problem.[3] Indeed, many deaths resulted from bold innovation efforts during the early years of cardiac surgery.[4] More recently, it became apparent that patients had experienced unanticipated complications following the use of novel vaginal mesh procedures for stress urinary incontinence; initially the innovation was considered to be safe and effective, however the potential for harm emerged after several years.[5]

There has been an increasing awareness in recent years of the need to improve the process of surgical innovation, to enhance its efficiency and transparency whilst minimising the risk of harm to patients.[6] Pharmacological innovation provides an appropriate analogy, as a well-defined phased approach for evaluating new drugs is already widely used.[7] Prior to the first use of a new drug in humans, its safety and pharmacological properties are studied in animals. The first use in humans then occurs during Phase I trials, which investigate safety and dosing in small numbers of healthy or diseased participants. In phase II trials, safety and pharmacological properties are explored in small numbers of diseased participants; they may provide preliminary evidence of efficacy and play an important role in preparing for Phase III evaluation. Phase III trials evaluate efficacy and identify common adverse events in larger

For peer review only

cohorts of participants. During phase IV, observational studies of large numbers of participants aim to detect uncommon adverse events and evaluate cost and effectiveness.

The phased clinical trials system represents a progressive and methodical approach to evaluating pharmacological innovations. Consequently, the risk of unmitigated harm is minimised, whilst the quality of evaluation is optimised.[7] Even beyond phased clinical trials, long-term risks and adverse events associated with new drugs are monitored using the "yellow card" scheme.[8] Unfortunately, no comparable methodical approach has been widely applied to surgical innovation, which has been generally unstandardised, under-governed, and marred by a lack of high-quality evaluation.[9] This can be attributed to the relative complexity of Invasive Procedures and Devices (IP/Ds) as interventions, making robust evaluation more difficult due to challenges such as lack of equipoise, standardisation of the intervention, learning curves, blinding, and selection of outcome measures.[10] These issues may be compounded by aspects of surgical culture and a lack of education about the importance of methodology amongst surgeons. Furthermore, it has been unclear when evaluation is necessary, due to difficulties in recognising when surgical innovation has occurred.[11]

The IDEAL (Idea, Development, Exploration, Assessment, Long-term study) Collaboration has promoted the concept of staged surgical innovation over the past decade. Its most recent iteration of the "IDEAL framework" described a sequence of five stages of innovation of invasive procedures; a separate "IDEAL-D" framework dealt with devices.[12, 13] The IDEAL framework begins with the first use in living humans at the "Idea" stage, and finishes with ongoing surveillance of the widely adopted and stable procedure during the fifth "Long-term study" stage. There is also a pre-clinical stage, which is not part of the framework itself, representing innovation prior to the first use in a living human. The framework provides recommendations regarding evaluation at each stage, with the aim of improving the quality of research in surgery. Despite further refinement of the framework and publication of practical

For peer review only

guidance, its adoption has been relatively slow, and many authors attempting to utilise the framework have failed to correctly apply its principles.[14, 15] An important reason may be that determining the stage of innovation of a procedure or device is often difficult.[15] To facilitate a methodical approach to evaluating surgical innovations, there is a need for a detailed insight into what stage of innovation means, and how it can be determined.

The aim of this study is to understand the concept of stage of innovation as reported in the literature. The objectives are: to identify existing approaches to evaluating surgical innovations, including methods for determining stage of innovation; and to identify related problems, with solutions or suggestions for further development.

METHODS AND ANALYSIS

A systematic review will be conducted and reported in accordance with the recommendations of the PRISMA (Preferred Reporting Items for Systematic reviews and Meta-Analyses) statement.[16] This protocol has been reported in accordance with the PRISMA-P statement, see online supplemental file 1. Any subsequent modifications will be described in the final report. This systematic review is registered with PROSPERO (CRD42021270812).

Search strategy

Published literature will be searched using the Ovid MEDLINE, Ovid Embase, CINAHL and INAHTA online bibliographic databases. The search will be developed with the assistance of an information specialist, using scoping searches to iteratively refine the strategy. The broad search concepts will be “stage of innovation”, “invasive procedures or devices”, and “guidance”. An example search for Ovid MEDLINE is presented in online supplemental file 2. The sensitivity of the search strategy will be verified by checking its ability to detect original reports from the IDEAL Collaboration, and refining the strategy if required to ensure they are detected.[12, 15, 17] Reference lists within selected articles from the search results will be

For peer review only

screened for other relevant articles. Experts in surgical innovation will be asked to suggest other relevant articles which may not have been detected by the search strategy.

Eligibility criteria

A search result will be eligible for inclusion if it: (i) describes an approach to evaluating surgical innovations, or; (ii) describes a problem with an approach, proposes a solution, or makes a recommendation for further development, or; (iii) describes a method for determining the stage of innovation of an IP/D, or; (iv) describes a problem with a method for determining stage of innovation, proposes a solution, or makes a recommendation for further development, or; (v) describes a property of an innovation which can infer its stage of innovation, or; (vi) defines a discrete stage of innovation, or; (vii) describes a potential source of stage-related information. This will include, but will not be limited to, the concept of stage of innovation as described by the IDEAL Collaboration.[12] Only innovation of IP/Ds will be eligible; innovation relating to other forms of therapy, such as pharmaceuticals, will be excluded. Invasive procedures will be defined according to the definition proposed by Cousins *et al*.[18] Devices will be restricted to a subset of medical devices as defined by the Medical Devices Regulations 2002 (SI 2002 No 618, as amended): general medical devices or active implantable medical devices which are both invasive and therapeutic.[19] This will exclude diagnostic medical devices. Approaches to surgical innovation may include descriptions, frameworks, models, classifications, typologies, guidelines, policies, or recommendations, which are intended to introduce structure into the process of evaluating surgical innovation. A wide range of article types will be eligible, including reports of primary and secondary research, guidelines, and opinion pieces. There will be no restrictions by date of publication. Conference abstracts and articles presented in languages other than English will be excluded because of the difficulties associated with data extraction.

For peer review only

Screening

Search results will be imported to EndNote X9 reference management software (Clarivate, 2013). Duplicate search results will be resolved using EndNote. Search results from published literature will first be screened by title and abstract. Full text articles will then be retrieved, read in full, and then screened. Screening of each title, abstract and full text will be undertaken by two independent assessors, with conflicts resolved by discussion. A third assessor will be consulted if a consensus is not reached, deciding the outcome by the majority opinion. The Rayyan web application will be used to facilitate screening.[20] Citations within eligible full text articles will be checked for any further potentially relevant articles.

Data extraction

Data extraction will be facilitated using the REDCap electronic data capture tool.[21] A data collection form will be pre-defined, piloted and iteratively refined using samples of articles returned by the search. Included articles will be read in full to identify relevant data, which may be extracted from any sections of the articles. Data will be extracted verbatim where possible.

Data will be extracted regarding the following:

- Article characteristics (e.g., title; year; authorship; type of medium; funding; country)
- Article type (e.g., letter; narrative review)
- Approaches to surgical innovation (e.g., name of systematic approach; original citation; form of presentation of approach; how it is used; appraisals, evaluations, opinions, critiques, recommendations, suggestions, or comments about the approach)
- Stage of innovation (e.g., meaning of stage of innovation; definitions of discrete stages; methods, guidance, advice, recommendations, descriptions, instructions, or explanations regarding how to determine stage of innovation; appraisals, evaluations,

For peer review only

opinions, critiques, recommendations, suggestions, or comments regarding any process for determining stage of innovation; properties or characteristics which might infer stage of innovation)

- Sources of stage-related information (e.g., where information about stage of innovation might be obtained; appraisals, evaluations, opinions, critiques, recommendations, suggestions, or comments about an information source; which properties or characteristics might be obtained from an information source; methods, guidance, advice, recommendations, descriptions, instructions, or explanations regarding how information regarding stage of innovation might be obtained from an information source)
- General comments (i.e., any other text which might be relevant to the aim of the study)

Data will be extracted independently from all eligible articles by both reviewers. The two sets of data will be compared for consistency; discrepancies will be resolved by discussion, or consultation with a third reviewer if required.

Data analysis

Descriptive data will be summarised in a table, including article characteristics and article types. A thematic analysis will be conducted for data relating to approaches to surgical innovation, stage of innovation, sources of stage-related information, and general comments, in accordance with the method described by Braun and Clarke.[22] Thematic analysis is appropriate for searching across a dataset for repeated patterns of meaning.[22] Therefore, it is well-suited for inductively extracting semantics and understanding regarding stage of innovation. The analysis comprises six steps: familiarisation with the data, generation of initial codes, searching for themes, reviewing themes, definition and naming of themes, and production of a report. The thematic analysis will be conducted by two reviewers, with frequent

For peer review only

comparisons of their codes and themes. Assessments of risk of study bias or methodological quality of included articles will not be appropriate for this study.

PATIENT AND PUBLIC INVOLVEMENT

This study will not directly involve patients or members of the public, due to its methodological focus.

ETHICS AND DISSEMINATION

Ethical approval will not be required as this will be secondary research utilising publicly available data. This systematic review will be published in a peer-reviewed journal and presented at appropriate conferences.

ACKNOWLEDGEMENTS

The authors wish to acknowledge the expert assistance provided by Catherine Borwick, University of Bristol, in developing the search strategy.

AUTHOR CONTRIBUTIONS

NSB and JMB conceived the initial idea for the study and initiated the study. DLS, DE, SC, KNLA, JMB, and NSB iterated the idea for the study and designed the study protocol. DLS prepared the initial and final drafts of the manuscript. DE, SC, KNLA, NSB and JMB critically revised the manuscript. All authors approved the final version of the manuscript.

FUNDING

This study was supported by the NIHR Biomedical Research Centre at University Hospitals Bristol and Weston NHS Foundation Trust and the University of Bristol. The views expressed

For peer review only

are those of the author(s) and not necessarily those of the NIHR or the Department of Health and Social Care. JMB is an NIHR Senior Investigator and NSB is an MRC Clinician Scientist.

COMPETING INTERESTS

The authors have no conflicts of interest to declare.

For peer review only

REFERENCES

1. Ozaki S, Kawase I, Yamashita H, et al. Midterm outcomes after aortic valve neocuspidization with glutaraldehyde-treated autologous pericardium. *J Thorac Cardiovasc Surg* 2018;155(6):2379-87.
2. Howard C, Jullian L, Joshi M, et al. TAVI and the future of aortic valve replacement. *J Card Surg* 2019;34(12):1577-90.
3. Birchley G, Ives J, Huxtable R, et al. Conceptualising Surgical Innovation: An Eliminativist Proposal. *Health Care Anal* 2020;28(1):73-97.
4. Hessel EA, 2nd. A Brief History of Cardiopulmonary Bypass. *Semin Cardiothorac Vasc Anesth* 2014;18(2):87-100.
5. Keltie K, Elneil S, Monga A, et al. Complications following vaginal mesh procedures for stress urinary incontinence: an 8 year study of 92,246 women. *Sci Rep* 2017;7(1):12015.
6. Karpowicz L, Bell E, Racine E. Ethics Oversight Mechanisms for Surgical Innovation: A Systematic and Comparative Review of Arguments. *J Empir Res Hum Res Ethics* 2016;11(2):135-64.
7. Umscheid CA, Margolis DJ, Grossman CE. Key concepts of clinical trials: a narrative review. *Postgrad Med* 2011;123(5):194-204.
8. Medicines and Healthcare products Regulatory Agency (MHRA). Yellow Card Scheme. https://yellowcard.mhra.gov.uk/the-yellow-card-scheme/. Accessed September 2021.
9. Barkun JS, Aronson JK, Feldman LS, et al. Evaluation and stages of surgical innovations. *Lancet* 2009;374(9695):1089-96.
10. McCulloch P, Taylor I, Sasako M, et al. Randomised trials in surgery: problems and possible solutions. *BMJ* 2002;324(7351):1448-51.
11. Blakely B, Selwood A, Rogers WA, et al. Macquarie Surgical Innovation Identification Tool (MSIIT): a study protocol for a usability and pilot test. *BMJ Open* 2016;6(11):e013704.
12. Hirst A, Philippou Y, Blazeby J, et al. No Surgical Innovation Without Evaluation: Evolution and Further Development of the IDEAL Framework and Recommendations. *Ann Surg* 2019;269(2):211-20.
13. Sedrakyan A, Campbell B, Merino JG, et al. IDEAL-D: a rational framework for evaluating and regulating the use of medical devices. *BMJ* 2016;353:i2372.
14. Khachane A, Philippou Y, Hirst A, et al. Appraising the uptake and use of the IDEAL Framework and Recommendations: A review of the literature. *Int J Surg* 2018;57:84-90.
15. Pennell CP, Hirst AD, Campbell WB, et al. Practical guide to the Idea, Development and Exploration stages of the IDEAL Framework and Recommendations. *Br J Surg* 2016;103(5):607-15.
16. Moher D, Liberati A, Tetzlaff J, et al. Preferred reporting items for systematic reviews and meta-analyses: the PRISMA statement. *PLoS Med* 2009;6(7):e1000097.
17. McCulloch P, Altman DG, Campbell WB, et al. No surgical innovation without evaluation: the IDEAL recommendations. *Lancet* 2009;374(9695):1105-12.
18. Cousins S, Blencowe NS, Blazeby JM. What is an invasive procedure? A definition to inform study design, evidence synthesis and research tracking. *BMJ Open* 2019;9(7):e028576.
19. The Medical Devices Regulations 2002. https://www.legislation.gov.uk/uksi/2002/618/pdfs/uksi_20020618_en.pdf. Accessed September 2021.
20. Ouzzani M, Hammady H, Fedorowicz Z, et al. Rayyan-a web and mobile app for systematic reviews. *Syst Rev* 2016;5(1):210.

For peer review only

21. Harris PA, Taylor R, Thielke R, et al. Research electronic data capture (REDCap)--a metadata-driven methodology and workflow process for providing translational research informatics support. *J Biomed Inform* 2009;42(2):377-81.
22. Braun V, Clarke V. Using thematic analysis in psychology. *Qualitative Research in Psychology* 2006;3(2):77-101.

For peer review only

PRISMA-P Checklist

Adapted from (1).

Section and topic	Item No.	Checklist item	Included
**Administrative info.**			
Title:			
Identification	1a	Identify the report as a protocol of a systematic review	✓
Update	1b	If the protocol is for an update of a previous systematic review, identify as such	n/a
Registration	2	If registered, provide the name of the registry (such as PROSPERO) and registration number	n/a
Authors:			
Contact	3a	Provide name, institutional affiliation, e-mail address of all protocol authors; provide physical mailing address of corresponding author	✓
Contributions	3b	Describe contributions of protocol authors and identify the guarantor of the review	✓
Amendments	4	If the protocol represents an amendment of a previously completed or published protocol, identify as such and list changes; otherwise, state plan for documenting important protocol amendments	✓
Support:			
Sources	5a	Indicate sources of financial or other support for the review	✓
Sponsor	5b	Provide name for the review funder and/or sponsor	✓
Role of sponsor or funder	5c	Describe roles of funder(s), sponsor(s), and/or institution(s), if any, in developing the protocol	n/a
**Introduction**			
Rationale	6	Describe the rationale for the review in the context of what is already known	✓
Objectives	7	Provide an explicit statement of the question(s) the review will address with reference to participants, interventions, comparators, and outcomes (PICO)	n/a
**Methods**			
Eligibility criteria	8	Specify the study characteristics (such as PICO, study design, setting, time frame) and report characteristics (such as years considered, language, publication status) to be used as criteria for eligibility for the review	✓

For peer review only

Information sources	9	Describe all intended information sources (such as electronic databases, contact with study authors, trial registers or other grey literature sources) with planned dates of coverage	✓
Search strategy	10	Present draft of search strategy to be used for at least one electronic database, including planned limits, such that it could be repeated	✓
Study records:			
Data management	11a	Describe the mechanism(s) that will be used to manage records and data throughout the review	✓
Selection process	11b	State the process that will be used for selecting studies (such as two independent reviewers) through each phase of the review (that is, screening, eligibility and inclusion in meta-analysis)	✓
Data collection process	11c	Describe planned method of extracting data from reports (such as piloting forms, done independently, in duplicate), any processes for obtaining and confirming data from investigators	✓
Data items	12	List and define all variables for which data will be sought (such as PICO items, funding sources), any pre-planned data assumptions and simplifications	✓
Outcomes and prioritization	13	List and define all outcomes for which data will be sought, including prioritization of main and additional outcomes, with rationale	n/a
Risk of bias in individual studies	14	Describe anticipated methods for assessing risk of bias of individual studies, including whether this will be done at the outcome or study level, or both; state how this information will be used in data synthesis	n/a
Data synthesis	15a	Describe criteria under which study data will be quantitatively synthesised	n/a
	15b	If data are appropriate for quantitative synthesis, describe planned summary measures, methods of handling data and methods of combining data from studies, including any planned exploration of consistency (such as I^2, Kendall's τ)	n/a
	15c	Describe any proposed additional analyses (such as sensitivity or subgroup analyses, meta-regression)	n/a
	15d	If quantitative synthesis is not appropriate, describe the type of summary planned	✓
Meta-bias(es)	16	Specify any planned assessment of meta-bias(es) (such as publication bias across studies, selective reporting within studies)	n/a
Confidence in cumulative evidence	17	Describe how the strength of the body of evidence will be assessed (such as GRADE)	n/a

1. Shamseer L, Moher D, Clarke M, Ghersi D, Liberati A, Petticrew M, et al. Preferred reporting items for systematic review and meta-analysis protocols (PRISMA-P) 2015: elaboration and explanation. BMJ. 2015;350:g7647.

For peer review only

Search strategy for Ovid MEDLINE

#	Searches
1	((stage or phase or framework) adj2 (IDEAL or development or innovat*)).ti,ab.
2	IDEAL-D.ti,ab,kf,hw.
3	1 or 2
4	(invasive or incision or cut or percutaneous or puncture or (natural adj1 orifice)).ti,ab,kf,hw.
5	(endoscop* or colonoscop* or gastroscop* or thoracoscop* or laparoscop* or arthroscop* or bronchoscop*).ti,ab,kf,hw.
6	(catheter or scalpel or surgery or surgical or operat* or interventional).ti,ab,kf,hw.
7	(device* or implant* or prosthe* or robot*).ti,ab,kf,hw.
8	exp Specialties, Surgical/ or exp Endoscopy/ or Radiology, Interventional/ or exp Surgical Procedures, Operative/ or exp "prostheses and implants"/
9	4 or 5 or 6 or 7 or 8
10	(guideline* or guidance or recommendation* or proposal* or regulation* or advice or framework or typology or model or algorithm or evaluat* or determin* or practical method* or method* or concept* or hinder* or hindrance* or difficult* or problem*).ti,ab.
11	exp Guidelines as Topic/ or exp Classification/
12	10 or 11
13	3 and 9 and 12
14	limit 13 to english language

For peer review only

BMJ Open

Understanding stage of innovation of invasive procedures and devices: protocol for a systematic review and thematic analysis

Journal:	*BMJ Open*
Manuscript ID	bmjopen-2021-057842.R1
Article Type:	Protocol
Date Submitted by the Author:	23-Dec-2021
Complete List of Authors:	Scroggie, Darren; NIHR Bristol Biomedical Research Centre; University Hospitals Bristol and Weston NHS Foundation Trust<br>Elliott, Daisy; NIHR Bristol Biomedical Research Centre<br>Cousins, Sian; NIHR Bristol Biomedical Research Centre<br>Avery, Kerry; NIHR Bristol Biomedical Research Centre<br>Blazeby, Jane; NIHR Bristol Biomedical Research Centre; University Hospitals Bristol and Weston NHS Foundation Trust<br>Blencowe, Natalie; NIHR Bristol Biomedical Research Centre; University Hospitals Bristol and Weston NHS Foundation Trust
**Primary Subject Heading**:	Surgery
Secondary Subject Heading:	Evidence based practice, Qualitative research
Keywords:	QUALITATIVE RESEARCH, Clinical governance < HEALTH SERVICES ADMINISTRATION & MANAGEMENT, STATISTICS & RESEARCH METHODS, SURGERY, THERAPEUTICS

SCHOLARONE™
Manuscripts

Understanding stage of innovation of invasive procedures and devices: protocol for a systematic review and thematic analysis

Darren L Scroggie [a,b] darren.scroggie@bristol.ac.uk
Daisy Elliott [a] daisy.elliott@bristol.ac.uk
Sian Cousins [a] sian.cousins@bristol.ac.uk
Kerry NL Avery [a] kerry.avery@bristol.ac.uk
Jane Blazeby [a,b] j.m.blazeby@bristol.ac.uk
Natalie S Blencowe [a,b] natalie.blencowe@bristol.ac.uk

Corresponding author:
Darren L Scroggie
Bristol Centre for Surgical Research
Bristol Medical School
Department of Population Health Sciences
Canynge Hall
39 Whatley Road
Bristol
BS8 2PS
United Kingdom
darren.scroggie@bristol.ac.uk

Guarantor:
Natalie S Blencowe
Bristol Centre for Surgical Research
Bristol Medical School
Department of Population Health Sciences
Canynge Hall
39 Whatley Road
Bristol
BS8 2PS
United Kingdom
natalie.blencowe@bristol.ac.uk

[a] National Institute for Health Research
Bristol and Weston Biomedical Research Centre
Bristol Centre for Surgical Research
Bristol Medical School
Department of Population Health Sciences
Canynge Hall
39 Whatley Road
Bristol
BS8 2PS
United Kingdom

[b] University Hospitals Bristol and Weston NHS Foundation Trust
Bristol
BS2 8HW
United Kingdom

Word count: 1950

For peer review only

ABSTRACT

Introduction

Surgical innovation has generally occurred in an unstandardised manner. This has led to unnecessary exposure of patients to harm, research waste, and an inadequate evidence base. The IDEAL (Idea, Development, Exploration, Assessment, Long-term follow-up) Collaboration provided a structured set of recommendations for evaluating surgical innovations based on their stage of innovation. Despite further refinements and guidance, adoption of the IDEAL recommendations has been slow; an important reason may be that determining the stage of innovation is often difficult. To facilitate evaluation of surgical innovations, there is a need for a detailed insight into what stage of innovation means, and how it can be determined. The aim of this study is to understand the concept of stage of innovation as reported in the literature.

Methods and analysis

A systematic review is being conducted. Ovid MEDLINE and Embase databases were searched from their inception until July 2021 using a strategy based upon the concepts of stage of innovation, invasive procedures or devices, and guidance. Articles were included if they described an approach to evaluating surgical innovations in stages, described a method for determining stage of innovation, described indicators of stage of innovation, defined stages, or described potential sources of stage-related information. Conference abstracts and non-English language articles were excluded. Other articles were detected from citations within included articles and suggestions from experts in surgical innovation. Data will be extracted regarding approaches to evaluating surgical innovations, methods for determining stage of innovation, indicators of stage of innovation, definitions of stages, and potential sources of stage-related

For peer review only

information. A thematic analysis will be conducted, and findings summarised in a narrative report.

Ethics and dissemination

Ethical approval will not be required. This systematic review will be published in a peer-reviewed journal and presented at appropriate conferences.

Registration

This systematic review is registered with PROSPERO (CRD42021270812).

For peer review only

ARTICLE SUMMARY

Strengths and limitations of this study

- A comprehensive search strategy and inclusive eligibility criteria will maximise the breadth of data obtained
- Thematic analysis is well-suited for searching for meaning and current understanding regarding abstract concepts such as stage of innovation
- An improved understanding of stage of innovation may support wider adoption of approaches to evaluating surgical innovations
- This study will be limited to articles written in the English language

For peer review only

INTRODUCTION

Progress in surgical practice has been driven by innovation. The continuous development and implementation of new ideas has led to the myriad surgical procedures in widespread use today. Innovation of surgical procedures has often been accompanied by innovation of related medical devices. For example, endoluminal techniques and deployable artificial heart valves have resulted in effective and safe treatment options for otherwise untreatable patient cohorts.[1] Whilst innovation aims to improve outcomes, it is associated with unknown risks and potential harm.[2] Many deaths resulted from bold innovation efforts during the early years of cardiac surgery.[3] More recently, it became apparent that patients had experienced unanticipated complications following the use of new vaginal mesh procedures for stress urinary incontinence.[4] Initially the innovation was considered to be safe and effective; the potential for harm was realised after several years.

The governance processes for medical devices and invasive procedures are different in the United Kingdom (UK). The regulation of medical devices is the responsibility of the Government via the Medicines and Healthcare products Regulatory Agency (MHRA).[5] Products which meet the legal definition of a medical device may be subject to a conformity assessment by a UK Approved Body, which considers scientific data, manufacturing processes and quality management. A UK Conformity Assessed (UKCA) certificate is issued if the checks are satisfactory. The National Institute for health and Care Excellence (NICE) considers the governance of new invasive procedures to be the responsibility of healthcare provider organisations.[6] NICE's Interventional Procedures Programme (IPP) recommends that National Health Service (NHS) providers should have governance structures to review, authorise and monitor the introduction of new procedures.[6] The Royal College of Surgeons of England (RCS) advocates the creation of local surgical innovation committees dedicated to this role.[7] These governance processes for new invasive procedures are not legal

For peer review only

requirements, and are not enforced by the Government. Furthermore, there has been a lack of clarity about when and how to implement governance mechanisms for new invasive procedures.[8]

Identifying when surgical innovation is occurring is often problematic, as the distinction between routine variation in surgical practice and innovation is not clear.[9] The lack of a clear definition of surgical innovation makes it difficult to understand when the governance processes for new procedures suggested by NICE and the RCS should be implemented. The Macquarie Surgical Innovation Identification Tool (MSIIT) was developed to help surgeons and healthcare providers recognise innovation prospectively, although its usefulness has not yet been established.[8, 10]

There has been increasing awareness of the need to improve the evaluation of surgical innovations.[11] Pharmacological innovation provides an appropriate comparator, as a well-defined phased approach for determining the safety and effectiveness of new drugs is widely used.[12] This approach minimises the risk of harm, whilst optimising the scientific quality of evidence.[12] Even beyond phased clinical trials, long-term risks, adverse events and rare outcomes associated with new drugs are monitored using the MHRA's "yellow card" scheme.[13] No comparable approach has been widely applied to surgical innovation, which has been generally unstandardised, under-governed, and marred by a lack of high-quality evaluation.[14, 15]

The IDEAL (Idea, Development, Exploration, Assessment, Long-term study) Collaboration has promoted the concept of staged surgical innovation over the past decade. Its most recent iteration of the "IDEAL framework" described a sequence of five stages of innovation of invasive procedures; a modification of the framework known as "IDEAL-D" dealt with devices.[16, 17] The IDEAL framework begins with the first use in living humans at the "Idea"

For peer review only

stage, and finishes with ongoing surveillance of the widely adopted and stable procedure during the fifth “Long-term study” stage. There is also a pre-clinical stage, which is not part of the framework itself, representing innovation prior to the first use in a living human. The framework provides recommendations regarding evaluation at each stage, with the aim of improving the quality of research in surgery. Despite further refinement of the framework and publication of practical guidance, its adoption has been relatively slow, and many authors attempting to utilise the framework have failed to correctly apply its principles.[18, 19] An important reason may be that determining the stage of innovation of a procedure or device is often difficult.[19] To facilitate a methodical approach to evaluating surgical innovations, there is a need for a detailed insight into what stage of innovation means, and how it can be determined.

The aim of this study is to understand the concept of stage of innovation as reported in the literature. The objectives are: to identify existing approaches to evaluating surgical innovations in stages, including methods for determining stage of innovation; and to identify related problems, with solutions or suggestions for further development.

METHODS AND ANALYSIS

A systematic review will be conducted and reported in accordance with the recommendations of the PRISMA (Preferred Reporting Items for Systematic reviews and Meta-Analyses) statement.[20] This protocol has been reported in accordance with the PRISMA-P statement, see online supplemental file 1.[21] Any subsequent modifications will be described in the final report. We anticipate completing the analysis by June 2022. This systematic review is registered with PROSPERO (CRD42021270812).

For peer review only

Search strategy

Published literature was searched using the Ovid MEDLINE and Embase online bibliographic databases. The search was developed with the assistance of an information specialist, using scoping searches to iteratively refine the strategy. The broad search concepts were stage of innovation, invasive procedures or devices, and guidance. The search strategies for Ovid MEDLINE and Embase are shown in online supplemental files 2 and 3. The sensitivity of the search strategy was verified by checking its ability to detect original reports from the IDEAL Collaboration, and refining the strategy as required to ensure they were detected.[16, 22] Reference lists within selected articles from the search results were screened for other relevant articles. Experts in surgical innovation will be asked to suggest other relevant articles which may not have been detected by the search strategy.

Eligibility criteria

A search result was eligible for inclusion if it: (i) described an approach to evaluating surgical innovations in stages, or; (ii) described a problem with an approach, proposed a solution, or made a recommendation for further development, or; (iii) described a method for determining the stage of innovation of an IP/D, or; (iv) described a problem with a method for determining stage of innovation, proposed a solution, or made a recommendation for further development, or; (v) described a property of an innovation which can infer its stage of innovation, or; (vi) defined a discrete stage of innovation, or; (vii) described a potential source of stage-related information. This included, but was not limited to, the concept of stage of innovation as described by the IDEAL Collaboration.[16] Only innovation of IP/Ds was eligible; innovation relating to other forms of therapy, such as pharmaceuticals, was excluded.

Invasive procedures were defined according to the definition proposed by Cousins *et al*: "*An invasive procedure is one where purposeful/deliberate access to the body is gained via an incision, percutaneous puncture, where instrumentation is used in addition to the puncture*

For peer review only

needle, or instrumentation via a natural orifice. It begins when entry to the body is gained and ends when the instrument is removed, and/or the skin is closed. Invasive procedures are performed by trained healthcare professionals using instruments, which include, but are not limited to, endoscopes, catheters, scalpels, scissors, devices and tubes. Where invasive procedures also involve the administration of a medicinal product, these could be categorised as being part of an 'invasive procedure' when operator skill is required for its administration within the body, that is, when an internal action is performed to administer the product or the product is administered to a targeted anatomical area … There are also procedures which involve operator skill to target something inside the body (eg, electromagnetic radiation in the eye) without an incision, percutaneous puncture or instrumentation via a natural orifice. These types of procedures do not fall within the definition of an invasive procedure."[23]

Devices were restricted to a subset of medical devices as defined by the Medical Devices Regulations 2002 (SI 2002 No 618, as amended): general medical devices or active implantable medical devices which were both invasive and therapeutic.[24] This excluded diagnostic medical devices. Approaches to surgical innovation included descriptions, frameworks, models, classifications, typologies, guidelines, policies, or recommendations, which were intended to introduce structure into the process of evaluating surgical innovation. A wide range of article types were eligible, including reports of primary and secondary research, guidelines, and opinion pieces. There were no restrictions by date of publication. Conference abstracts and articles presented in languages other than English were excluded because of the difficulties associated with data extraction.

Screening

Search results were imported to EndNote X9 reference management software (Clarivate, 2013). Duplicate search results were resolved using EndNote. Search results from published literature were first screened by title and abstract. Full text articles were then retrieved, read in full, and

For peer review only

screened. Screening of each title, abstract and full text was undertaken by two independent assessors, with conflicts resolved by discussion. A third assessor was consulted if a consensus was not reached, deciding the outcome by the majority opinion. The Rayyan web application was used to facilitate screening.[25] Citations within eligible full text articles were checked for any further potentially relevant articles.

Data extraction

Data extraction will be facilitated using the REDCap electronic data capture tool.[26] A data collection form will be pre-defined, piloted and iteratively refined using samples of articles returned by the search. Included articles will be read in full to identify relevant data, which may be extracted from any sections of the articles. Data will be extracted verbatim where possible.

Data will be extracted regarding the following:

- Article characteristics (e.g., title; year; authorship; type of medium; funding; country)
- Article type (e.g., letter; narrative review)
- Approaches to evaluating surgical innovations (e.g., name of approach; original citation; form of presentation of approach; how it is used; appraisals, evaluations, opinions, critiques, recommendations, suggestions, or comments about the approach)
- Stage of innovation (e.g., meaning of stage of innovation; definitions of discrete stages; methods, guidance, advice, recommendations, descriptions, instructions, or explanations regarding how to determine stage of innovation; appraisals, evaluations, opinions, critiques, recommendations, suggestions, or comments regarding any process for determining stage of innovation; properties or characteristics which might infer stage of innovation)

For peer review only

- Sources of stage-related information (e.g., where information about stage of innovation might be obtained; appraisals, evaluations, opinions, critiques, recommendations, suggestions, or comments about an information source; which properties or characteristics might be obtained from an information source; methods, guidance, advice, recommendations, descriptions, instructions, or explanations regarding how information regarding stage of innovation might be obtained from an information source)
- General comments (i.e., any other text which might be relevant to the aim of the study)

Data will be extracted independently from all eligible articles by both reviewers. The two sets of data will be compared for consistency; discrepancies will be resolved by discussion, or consultation with a third reviewer if required.

Data analysis

Descriptive data will be summarised in a table, including article characteristics and article types. A thematic analysis will be conducted for data relating to approaches to evaluating surgical innovations, stages of innovation, sources of stage-related information, and general comments, in accordance with the method described by Braun and Clarke.[27] Thematic analysis is appropriate for searching across a dataset for repeated patterns of meaning.[27] It is well-suited for inductively extracting semantics and understanding regarding stage of innovation. The analysis comprises six steps: familiarisation with the data, generation of initial codes, searching for themes, reviewing themes, definition and naming of themes, and production of a report. The thematic analysis will be conducted by two reviewers, with frequent comparisons of their codes and themes. Assessments of risk of study bias or methodological quality of included articles will not be appropriate for this study.

For peer review only

PATIENT AND PUBLIC INVOLVEMENT

This study will not directly involve patients or members of the public, due to its methodological focus.

ETHICS AND DISSEMINATION

Ethical approval will not be required as this will be secondary research utilising publicly available data. This systematic review will be published in a peer-reviewed journal and presented at appropriate conferences.

ACKNOWLEDGEMENTS

The authors wish to acknowledge the expert assistance provided by Catherine Borwick, University of Bristol, in developing the search strategy.

AUTHOR CONTRIBUTIONS

NSB and JMB conceived the initial idea for the study and initiated the study. DLS, DE, SC, KNLA, JMB, and NSB iterated the idea for the study and designed the study protocol. DLS prepared the initial and final drafts of the manuscript. DE, SC, KNLA, NSB and JMB critically revised the manuscript. All authors approved the final version of the manuscript.

FUNDING

This study was supported by the NIHR Biomedical Research Centre at University Hospitals Bristol and Weston NHS Foundation Trust and the University of Bristol. The views expressed are those of the author(s) and not necessarily those of the NIHR or the Department of Health and Social Care. JMB is an NIHR Senior Investigator and NSB is an MRC Clinician Scientist.

For peer review only

COMPETING INTERESTS

The authors have no conflicts of interest to declare.

For peer review only

REFERENCES

1. Howard C, Jullian L, Joshi M, et al. TAVI and the future of aortic valve replacement. *J Card Surg* 2019;34(12):1577-90.

2. Birchley G, Ives J, Huxtable R, et al. Conceptualising Surgical Innovation: An Eliminativist Proposal. *Health Care Anal* 2020;28(1):73-97.

3. Hessel EA, 2nd. A Brief History of Cardiopulmonary Bypass. *Semin Cardiothorac Vasc Anesth* 2014;18(2):87-100.

4. Keltie K, Elneil S, Monga A, et al. Complications following vaginal mesh procedures for stress urinary incontinence: an 8 year study of 92,246 women. *Sci Rep* 2017;7(1):12015.

5. Medicines and Heathcare products Regulatory Agency (MHRA). Medical devices: how to comply with the legal requirements in Great Britain. https://www.gov.uk/guidance/medical-devices-how-to-comply-with-the-legal-requirements#classification-devices (accessed 20th December, 2021).

6. National Institute for Health and Care Excellence (NICE). The National Institute for Health and Care Excellence (NICE) Interventional Procedures Programme. Summary of requirements for the NHS and clinicians. 2017. https://www.nice.org.uk/Media/Default/About/what-we-do/our-programmes/interventional-procedures/Using-new-IPs-requirements-NHS-and-clinicians.pdf (accessed 20th December, 2021).

7. Royal College of Surgeons of England (RCS). Surgical innovation, new techniques and technologies: A guide to good practice. 2019. https://www.rcseng.ac.uk/-/media/files/rcs/standards-and-research/standards-and-policy/good-practice-guides/old-documents/surgical-innovation-new-techniques-and-technologies.pdf (accessed 22nd September, 2021).

8. Hutchison K, Rogers W, Eyers A, et al. Getting Clearer About Surgical Innovation: A New Definition and a New Tool to Support Responsible Practice. *Annals of surgery* 2015;262(6):949-54.

9. Rogers WA, Lotz M, Hutchison K, et al. Identifying surgical innovation: a qualitative study of surgeons' views. *Ann Surg* 2014;259(2):273-8.

10. Blakely B, Selwood A, Rogers WA, et al. Macquarie Surgical Innovation Identification Tool (MSIIT): a study protocol for a usability and pilot test. *BMJ Open* 2016;6(11):e013704.

11. Karpowicz L, Bell E, Racine E. Ethics Oversight Mechanisms for Surgical Innovation: A Systematic and Comparative Review of Arguments. *J Empir Res Hum Res Ethics* 2016;11(2):135-64.

12. Umscheid CA, Margolis DJ, Grossman CE. Key concepts of clinical trials: a narrative review. *Postgrad Med* 2011;123(5):194-204.

13. Medicines and Heathcare products Regulatory Agency (MHRA). Yellow Card Scheme. https://yellowcard.mhra.gov.uk/the-yellow-card-scheme/ (accessed 22nd September, 2021).

14. Barkun JS, Aronson JK, Feldman LS, et al. Evaluation and stages of surgical innovations. *Lancet* 2009;374(9695):1089-96.

For peer review only

15. Skivington K, Matthews L, Simpson SA, et al. A new framework for developing and evaluating complex interventions: update of Medical Research Council guidance. *BMJ* 2021;374:n2061.

16. Hirst A, Philippou Y, Blazeby J, et al. No Surgical Innovation Without Evaluation: Evolution and Further Development of the IDEAL Framework and Recommendations. *Ann Surg* 2019;269(2):211-20.

17. Sedrakyan A, Campbell B, Merino JG, et al. IDEAL-D: a rational framework for evaluating and regulating the use of medical devices. *Bmj* 2016;353:i2372.

18. Khachane A, Philippou Y, Hirst A, et al. Appraising the uptake and use of the IDEAL Framework and Recommendations: A review of the literature. *Int J Surg* 2018;57:84-90.

19. Pennell CP, Hirst AD, Campbell WB, et al. Practical guide to the Idea, Development and Exploration stages of the IDEAL Framework and Recommendations. *Br J Surg* 2016;103(5):607-15.

20. Moher D, Liberati A, Tetzlaff J, et al. Preferred reporting items for systematic reviews and meta-analyses: the PRISMA statement. *PLoS Med* 2009;6(7):e1000097.

21. Shamseer L, Moher D, Clarke M, et al. Preferred reporting items for systematic review and meta-analysis protocols (PRISMA-P) 2015: elaboration and explanation. *Bmj* 2015;350:g7647.

22. McCulloch P, Altman DG, Campbell WB, et al. No surgical innovation without evaluation: the IDEAL recommendations. *Lancet* 2009;374(9695):1105-12.

23. Cousins S, Blencowe NS, Blazeby JM. What is an invasive procedure? A definition to inform study design, evidence synthesis and research tracking. *BMJ Open* 2019;9(7):e028576.

24. The Medical Devices Regulations 2002. https://www.legislation.gov.uk/uksi/2002/618/pdfs/uksi_20020618_en.pdf (accessed 22nd September, 2021).

25. Ouzzani M, Hammady H, Fedorowicz Z, et al. Rayyan-a web and mobile app for systematic reviews. *Syst Rev* 2016;5(1):210.

26. Harris PA, Taylor R, Thielke R, et al. Research electronic data capture (REDCap)--a metadata-driven methodology and workflow process for providing translational research informatics support. *J Biomed Inform* 2009;42(2):377-81.

27. Braun V, Clarke V. Using thematic analysis in psychology. *Qualitative Research in Psychology* 2006;3(2):77-101.

For peer review only

PRISMA-P Checklist

Adapted from [1].

Section and topic	Item No.	Checklist item	Included
**Administrative info.**			
Title:			
Identification	1a	Identify the report as a protocol of a systematic review	✓
Update	1b	If the protocol is for an update of a previous systematic review, identify as such	n/a
Registration	2	If registered, provide the name of the registry (such as PROSPERO) and registration number	n/a
Authors:			
Contact	3a	Provide name, institutional affiliation, e-mail address of all protocol authors; provide physical mailing address of corresponding author	✓
Contributions	3b	Describe contributions of protocol authors and identify the guarantor of the review	✓
Amendments	4	If the protocol represents an amendment of a previously completed or published protocol, identify as such and list changes; otherwise, state plan for documenting important protocol amendments	✓
Support:			
Sources	5a	Indicate sources of financial or other support for the review	✓
Sponsor	5b	Provide name for the review funder and/or sponsor	✓
Role of sponsor or funder	5c	Describe roles of funder(s), sponsor(s), and/or institution(s), if any, in developing the protocol	n/a
**Introduction**			
Rationale	6	Describe the rationale for the review in the context of what is already known	✓
Objectives	7	Provide an explicit statement of the question(s) the review will address with reference to participants, interventions, comparators, and outcomes (PICO)	n/a
**Methods**			
Eligibility criteria	8	Specify the study characteristics (such as PICO, study design, setting, time frame) and report characteristics (such as years considered, language, publication status) to be used as criteria for eligibility for the review	✓

For peer review only

Information sources	9	Describe all intended information sources (such as electronic databases, contact with study authors, trial registers or other grey literature sources) with planned dates of coverage	✓
Search strategy	10	Present draft of search strategy to be used for at least one electronic database, including planned limits, such that it could be repeated	✓
Study records:			
Data management	11a	Describe the mechanism(s) that will be used to manage records and data throughout the review	✓
Selection process	11b	State the process that will be used for selecting studies (such as two independent reviewers) through each phase of the review (that is, screening, eligibility and inclusion in meta-analysis)	✓
Data collection process	11c	Describe planned method of extracting data from reports (such as piloting forms, done independently, in duplicate), any processes for obtaining and confirming data from investigators	✓
Data items	12	List and define all variables for which data will be sought (such as PICO items, funding sources), any pre-planned data assumptions and simplifications	✓
Outcomes and prioritization	13	List and define all outcomes for which data will be sought, including prioritization of main and additional outcomes, with rationale	n/a
Risk of bias in individual studies	14	Describe anticipated methods for assessing risk of bias of individual studies, including whether this will be done at the outcome or study level, or both; state how this information will be used in data synthesis	n/a
Data synthesis	15a	Describe criteria under which study data will be quantitatively synthesised	n/a
	15b	If data are appropriate for quantitative synthesis, describe planned summary measures, methods of handling data and methods of combining data from studies, including any planned exploration of consistency (such as I^2, Kendall's τ)	n/a
	15c	Describe any proposed additional analyses (such as sensitivity or subgroup analyses, meta-regression)	n/a
	15d	If quantitative synthesis is not appropriate, describe the type of summary planned	✓
Meta-bias(es)	16	Specify any planned assessment of meta-bias(es) (such as publication bias across studies, selective reporting within studies)	n/a
Confidence in cumulative evidence	17	Describe how the strength of the body of evidence will be assessed (such as GRADE)	n/a

1. Shamseer L, Moher D, Clarke M, Ghersi D, Liberati A, Petticrew M, et al. Preferred reporting items for systematic review and meta-analysis protocols (PRISMA-P) 2015: elaboration and explanation. BMJ. 2015;350:g7647.

For peer review only

Search Strategy for Ovid MEDLINE

	Searches
1	((stage or phase or framework) adj2 (IDEAL or development or innovat*)).ti,ab.
2	IDEAL-D.ti,ab,kf,hw.
3	1 or 2
4	(invasive or incision or cut or percutaneous or puncture or (natural adj1 orifice)).ti,ab,kf,hw.
5	(endoscop* or colonoscop* or gastroscop* or thoracoscop* or laparoscop* or arthroscop* or bronchoscop*).ti,ab,kf,hw.
6	(catheter or scalpel or surgery or surgical or operat* or interventional).ti,ab,kf,hw.
7	(device* or implant* or prosthe* or robot*).ti,ab,kf,hw.
8	exp Specialties, Surgical/ or exp Endoscopy/ or Radiology, Interventional/ or exp Surgical Procedures, Operative/ or exp "prostheses and implants"/
9	4 or 5 or 6 or 7 or 8
10	(guideline* or guidance or recommendation* or proposal* or regulation* or advice or framework or typology or model or algorithm or evaluat* or determin* or practical method* or method* or concept* or hinder* or hindrance* or difficult* or problem*).ti,ab.
11	exp Guidelines as Topic/ or exp Classification/
12	10 or 11
13	3 and 9 and 12
14	limit 13 to english language

For peer review only

Search Strategy for Embase

	Searches
1.	((stage or phase or framework) adj2 (IDEAL or development or innovat*)).ti,ab.
2.	IDEAL-D.ti,ab.
3.	1 or 2
4.	(invasive or incision or cut or percutaneous or puncture or (natural adj1 orifice)).ti,ab,tw.
5.	(endoscop* or colonoscop* or gastroscop* or thoracoscop* or laparoscop* or arthroscop* or bronchoscop*).ti,ab,tw.
6.	(catheter or scalpel or surgery or surgical or operat* or interventional).ti,ab,tw.
7.	(device* or implant* or prosthe* or robot*).ti,ab,tw.
8.	exp surgery/ or exp endoscopy/ or interventional radiology/ or exp "prostheses and orthoses"/
9.	4 or 5 or 6 or 7 or 8
10.	(guideline* or guidance or recommendation* or proposal* or regulation* or advice or framework or typology or model or algorithm or evaluat* or determin* or practical method* or method* or concept* or hinder* or hindrance* or difficult* or problem*).ti,ab.
11.	exp practice guideline/ or classification/
12.	10 or 11
13.	3 and 9 and 12
14.	limit 13 to english language

For peer review only

BMJ Open

Understanding stage of innovation of invasive procedures and devices: protocol for a systematic review and thematic analysis

Journal:	*BMJ Open*
Manuscript ID	bmjopen-2021-057842.R2
Article Type:	Protocol
Date Submitted by the Author:	21-Jan-2022
Complete List of Authors:	Scroggie, Darren; NIHR Bristol Biomedical Research Centre; University Hospitals Bristol and Weston NHS Foundation Trust<br>Elliott, Daisy; NIHR Bristol Biomedical Research Centre<br>Cousins, Sian; NIHR Bristol Biomedical Research Centre<br>Avery, Kerry; NIHR Bristol Biomedical Research Centre<br>Blazeby, Jane; NIHR Bristol Biomedical Research Centre; University Hospitals Bristol and Weston NHS Foundation Trust<br>Blencowe, Natalie; NIHR Bristol Biomedical Research Centre; University Hospitals Bristol and Weston NHS Foundation Trust
**Primary Subject Heading**:	Surgery
Secondary Subject Heading:	Evidence based practice, Qualitative research
Keywords:	QUALITATIVE RESEARCH, Clinical governance < HEALTH SERVICES ADMINISTRATION & MANAGEMENT, STATISTICS & RESEARCH METHODS, SURGERY, THERAPEUTICS

SCHOLARONE™
Manuscripts

Understanding stage of innovation of invasive procedures and devices: protocol for a systematic review and thematic analysis

Darren L Scroggie [a,b] darren.scroggie@bristol.ac.uk
Daisy Elliott [a] daisy.elliott@bristol.ac.uk
Sian Cousins [a] sian.cousins@bristol.ac.uk
Kerry NL Avery [a] kerry.avery@bristol.ac.uk
Jane Blazeby [a,b] j.m.blazeby@bristol.ac.uk
Natalie S Blencowe [a,b] natalie.blencowe@bristol.ac.uk

Corresponding author:
Darren L Scroggie
Bristol Centre for Surgical Research
Bristol Medical School
Department of Population Health Sciences
Canynge Hall
39 Whatley Road
Bristol
BS8 2PS
United Kingdom
darren.scroggie@bristol.ac.uk

Guarantor:
Natalie S Blencowe
Bristol Centre for Surgical Research
Bristol Medical School
Department of Population Health Sciences
Canynge Hall
39 Whatley Road
Bristol
BS8 2PS
United Kingdom
natalie.blencowe@bristol.ac.uk

[a] National Institute for Health Research
Bristol and Weston Biomedical Research Centre
Bristol Centre for Surgical Research
Bristol Medical School
Department of Population Health Sciences
Canynge Hall
39 Whatley Road
Bristol
BS8 2PS
United Kingdom

[b] University Hospitals Bristol and Weston NHS Foundation Trust
Bristol
BS2 8HW
United Kingdom

Word count: 1950

For peer review only

ABSTRACT

Introduction

Surgical innovation has generally occurred in an unstandardised manner. This has led to unnecessary exposure of patients to harm, research waste, and inadequate evidence. The IDEAL (Idea, Development, Exploration, Assessment, Long-term follow-up) Collaboration provided a set of recommendations for evaluating surgical innovations based on their stage of innovation. Despite further refinements and guidance, adoption of the IDEAL recommendations has been slow; an important reason may be that determining the stage of innovation is often difficult. To facilitate evaluation of surgical innovations, there is a need for a detailed insight into what stage of innovation means, and how it can be determined. The aim of this study is to understand the concept of stage of innovation as reported in the literature.

Methods and analysis

A systematic review is being conducted. Ovid MEDLINE and Embase databases were searched from their inception until July 2021 using an iteratively developed strategy based upon the concepts of stage of innovation, invasive procedures or devices, and guidance. Articles were included if they described an approach to evaluating surgical innovations in stages, described a method for determining stage of innovation, described indicators of stage of innovation, defined stages, or described potential sources of stage-related information. Conference abstracts and non-English language articles were excluded. Other articles were detected from citations within included articles and suggestions from experts in surgical innovation. Data will be extracted regarding approaches to evaluating surgical innovations, methods for determining stage of innovation, indicators of stage of innovation, definitions of stages, and potential sources of stage-related information. A thematic analysis will be conducted, and findings summarised in a narrative report.

Ethics and dissemination

Ethical approval will not be required. This systematic review will be published in a peer-reviewed journal and presented at appropriate conferences.

Registration

This systematic review is registered with PROSPERO (CRD42021270812).

For peer review only

ARTICLE SUMMARY

Strengths and limitations of this study

- A comprehensive search strategy and inclusive eligibility criteria will maximise the breadth of data obtained
- Thematic analysis is well-suited for searching for meaning and current understanding regarding abstract concepts such as stage of innovation
- An improved understanding of stage of innovation may support wider adoption of approaches to evaluating surgical innovations
- This study will be limited to articles written in the English language

For peer review only

INTRODUCTION

Progress in surgical practice has been driven by innovation. The continuous development and implementation of new ideas has led to the myriad surgical procedures in widespread use today. Innovation of surgical procedures has often been accompanied by innovation of related medical devices. For example, endoluminal techniques and deployable artificial heart valves have resulted in effective and safe treatment options for otherwise untreatable patient cohorts.[1] Whilst innovation aims to improve outcomes, it is associated with unknown risks and potential harm.[2] Many deaths resulted from bold innovation efforts during the early years of cardiac surgery.[3] More recently, it became apparent that patients had experienced unanticipated complications following the use of new vaginal mesh procedures for stress urinary incontinence.[4] Initially the innovation was considered to be safe and effective; the potential for harm was realised after several years.

The governance processes for medical devices and invasive procedures are different in the United Kingdom (UK). The regulation of medical devices is the responsibility of the Government via the Medicines and Healthcare products Regulatory Agency (MHRA).[5] Products which meet the legal definition of a medical device may be subject to a conformity assessment by a UK Approved Body, which considers scientific data, manufacturing processes and quality management. A UK Conformity Assessed (UKCA) certificate is issued if the checks are satisfactory. The National Institute for health and Care Excellence (NICE) considers the governance of new invasive procedures to be the responsibility of healthcare provider organisations.[6] NICE's Interventional Procedures Programme (IPP) recommends that National Health Service (NHS) providers should have governance structures to review, authorise and monitor the introduction of new procedures.[6] The Royal College of Surgeons of England (RCS) advocates the creation of local surgical innovation committees dedicated to this role.[7] These governance processes for new invasive procedures are not legal

For peer review only

requirements, and are not enforced by the Government. Furthermore, there has been a lack of clarity about when and how to implement governance mechanisms for new invasive procedures.[8]

Identifying when surgical innovation is occurring is often problematic, as the distinction between routine variation in surgical practice and innovation is not clear.[9] The lack of a clear definition of surgical innovation makes it difficult to understand when the governance processes for new procedures suggested by NICE and the RCS should be implemented. The Macquarie Surgical Innovation Identification Tool (MSIIT) was developed to help surgeons and healthcare providers recognise innovation prospectively, although its usefulness has not yet been established.[8, 10]

There has been increasing awareness of the need to improve the evaluation of surgical innovations.[11] Pharmacological innovation provides an appropriate comparator, as a well-defined phased approach for determining the safety and effectiveness of new drugs is widely used.[12] This approach minimises the risk of harm, whilst optimising the scientific quality of evidence.[12] Even beyond phased clinical trials, long-term risks, adverse events and rare outcomes associated with new drugs are monitored using the MHRA's "yellow card" scheme.[13] No comparable approach has been widely applied to surgical innovation, which has been generally unstandardised, under-governed, and marred by a lack of high-quality evaluation.[14, 15]

The IDEAL (Idea, Development, Exploration, Assessment, Long-term study) Collaboration has promoted the concept of staged surgical innovation over the past decade. Its most recent iteration of the "IDEAL framework" described a sequence of five stages of innovation of invasive procedures; a modification of the framework known as "IDEAL-D" dealt with devices.[16, 17] The IDEAL framework begins with the first use in living humans at the "Idea"

For peer review only

stage, and finishes with ongoing surveillance of the widely adopted and stable procedure during the fifth "Long-term study" stage. There is also a pre-clinical stage, which is not part of the framework itself, representing innovation prior to the first use in a living human. The framework provides recommendations regarding evaluation at each stage, with the aim of improving the quality of research in surgery. Despite further refinement of the framework and publication of practical guidance, its adoption has been relatively slow, and many authors attempting to utilise the framework have failed to correctly apply its principles.[18, 19] An important reason may be that determining the stage of innovation of a procedure or device is often difficult.[19] To facilitate a methodical approach to evaluating surgical innovations, there is a need for a detailed insight into what stage of innovation means, and how it can be determined.

The aim of this study is to understand the concept of stage of innovation as reported in the literature. The objectives are: to identify existing approaches to evaluating surgical innovations in stages, including methods for determining stage of innovation; and to identify related problems, with solutions or suggestions for further development.

METHODS AND ANALYSIS

A systematic review will be conducted and reported in accordance with the recommendations of the PRISMA (Preferred Reporting Items for Systematic reviews and Meta-Analyses) statement.[20] This protocol has been reported in accordance with the PRISMA-P statement, see online supplemental file 1.[21] Any subsequent modifications will be described in the final report. We anticipate completing the analysis by June 2022. This systematic review is registered with PROSPERO (CRD42021270812).

For peer review only

Search strategy

Published literature was searched using the Ovid MEDLINE and Embase online bibliographic databases. The search was developed with the assistance of an information specialist, using scoping searches to iteratively refine the strategy. The broad search concepts were stage of innovation, invasive procedures or devices, and guidance. The search strategies for Ovid MEDLINE and Embase are shown in online supplemental files 2 and 3. The sensitivity of the search strategy was verified by checking its ability to detect original reports from the IDEAL Collaboration, and refining the strategy as required to ensure they were detected.[16, 22] Reference lists within selected articles from the search results were screened for other relevant articles. Experts in surgical innovation will be asked to suggest other relevant articles which may not have been detected by the search strategy.

Eligibility criteria

A search result was eligible for inclusion if it: (i) described an approach to evaluating surgical innovations in stages, or; (ii) described a problem with an approach, proposed a solution, or made a recommendation for further development, or; (iii) described a method for determining the stage of innovation of an IP/D, or; (iv) described a problem with a method for determining stage of innovation, proposed a solution, or made a recommendation for further development, or; (v) described a property of an innovation which can infer its stage of innovation, or; (vi) defined a discrete stage of innovation, or; (vii) described a potential source of stage-related information. This included, but was not limited to, the concept of stage of innovation as described by the IDEAL Collaboration.[16] Only innovation of IP/Ds was eligible; innovation relating to other forms of therapy, such as pharmaceuticals, was excluded.

Invasive procedures were defined according to the definition proposed by Cousins *et al*: "*An invasive procedure is one where purposeful/deliberate access to the body is gained via an incision, percutaneous puncture, where instrumentation is used in addition to the puncture*

For peer review only

needle, or instrumentation via a natural orifice. It begins when entry to the body is gained and ends when the instrument is removed, and/or the skin is closed. Invasive procedures are performed by trained healthcare professionals using instruments, which include, but are not limited to, endoscopes, catheters, scalpels, scissors, devices and tubes. Where invasive procedures also involve the administration of a medicinal product, these could be categorised as being part of an 'invasive procedure' when operator skill is required for its administration within the body, that is, when an internal action is performed to administer the product or the product is administered to a targeted anatomical area … There are also procedures which involve operator skill to target something inside the body (eg, electromagnetic radiation in the eye) without an incision, percutaneous puncture or instrumentation via a natural orifice. These types of procedures do not fall within the definition of an invasive procedure."[23]

Devices were restricted to a subset of medical devices as defined by the Medical Devices Regulations 2002 (SI 2002 No 618, as amended): general medical devices or active implantable medical devices which were both invasive and therapeutic.[24] This excluded diagnostic medical devices. Approaches to surgical innovation included descriptions, frameworks, models, classifications, typologies, guidelines, policies, or recommendations, which were intended to introduce structure into the process of evaluating surgical innovation. A wide range of article types were eligible, including reports of primary and secondary research, guidelines, and opinion pieces. There were no restrictions by date of publication. Conference abstracts and articles presented in languages other than English were excluded because of the difficulties associated with data extraction.

Screening

Search results were imported to EndNote X9 reference management software (Clarivate, 2013). Duplicate search results were resolved using EndNote. Search results from published literature were first screened by title and abstract. Full text articles were then retrieved, read in full, and

For peer review only

screened. Screening of each title, abstract and full text was undertaken by two independent assessors, with conflicts resolved by discussion. A third assessor was consulted if a consensus was not reached, deciding the outcome by the majority opinion. The Rayyan web application was used to facilitate screening.[25] Citations within eligible full text articles were checked for any further potentially relevant articles.

Data extraction

Data extraction will be facilitated using the REDCap electronic data capture tool.[26] A data collection form will be pre-defined, piloted and iteratively refined using samples of articles returned by the search. Included articles will be read in full to identify relevant data, which may be extracted from any sections of the articles. Data will be extracted verbatim where possible.

Data will be extracted regarding the following:

- Article characteristics (e.g., title; year; authorship; type of medium; funding; country)
- Article type (e.g., letter; narrative review)
- Approaches to evaluating surgical innovations (e.g., name of approach; original citation; form of presentation of approach; how it is used; appraisals, evaluations, opinions, critiques, recommendations, suggestions, or comments about the approach)
- Stage of innovation (e.g., meaning of stage of innovation; definitions of discrete stages; methods, guidance, advice, recommendations, descriptions, instructions, or explanations regarding how to determine stage of innovation; appraisals, evaluations, opinions, critiques, recommendations, suggestions, or comments regarding any process for determining stage of innovation; properties or characteristics which might infer stage of innovation)

For peer review only

- Sources of stage-related information (e.g., where information about stage of innovation might be obtained; appraisals, evaluations, opinions, critiques, recommendations, suggestions, or comments about an information source; which properties or characteristics might be obtained from an information source; methods, guidance, advice, recommendations, descriptions, instructions, or explanations regarding how information regarding stage of innovation might be obtained from an information source)
- General comments (i.e., any other text which might be relevant to the aim of the study)

Data will be extracted independently from all eligible articles by both reviewers. The two sets of data will be compared for consistency; discrepancies will be resolved by discussion, or consultation with a third reviewer if required.

Data analysis

Descriptive data will be summarised in a table, including article characteristics and article types. A thematic analysis will be conducted for data relating to approaches to evaluating surgical innovations, stages of innovation, sources of stage-related information, and general comments, in accordance with the method described by Braun and Clarke.[27] Thematic analysis is appropriate for searching across a dataset for repeated patterns of meaning.[27] It is well-suited for inductively extracting semantics and understanding regarding stage of innovation. The analysis comprises six steps: familiarisation with the data, generation of initial codes, searching for themes, reviewing themes, definition and naming of themes, and production of a report. The thematic analysis will be conducted by two reviewers, with frequent comparisons of their codes and themes. Assessments of risk of study bias or methodological quality of included articles will not be appropriate for this study.

For peer review only

PATIENT AND PUBLIC INVOLVEMENT

This study will not directly involve patients or members of the public, due to its methodological focus.

ETHICS AND DISSEMINATION

Ethical approval will not be required as this will be secondary research utilising publicly available data. This systematic review will be published in a peer-reviewed journal and presented at appropriate conferences.

ACKNOWLEDGEMENTS

The authors wish to acknowledge the expert assistance provided by Catherine Borwick, University of Bristol, in developing the search strategy.

AUTHOR CONTRIBUTIONS

NSB and JMB conceived the initial idea for the study and initiated the study. DLS, DE, SC, KNLA, JMB, and NSB iterated the idea for the study and designed the study protocol. DLS prepared the initial and final drafts of the manuscript. DE, SC, KNLA, NSB and JMB critically revised the manuscript. All authors approved the final version of the manuscript.

FUNDING

This study was supported by the NIHR Biomedical Research Centre at University Hospitals Bristol and Weston NHS Foundation Trust and the University of Bristol. The views expressed are those of the author(s) and not necessarily those of the NIHR or the Department of Health and Social Care. JMB is an NIHR Senior Investigator and NSB is an MRC Clinician Scientist.

For peer review only

COMPETING INTERESTS

The authors have no conflicts of interest to declare.

For peer review only

REFERENCES

1. Howard C, Jullian L, Joshi M, et al. TAVI and the future of aortic valve replacement. *J Card Surg* 2019;34(12):1577-90.

2. Birchley G, Ives J, Huxtable R, et al. Conceptualising Surgical Innovation: An Eliminativist Proposal. *Health Care Anal* 2020;28(1):73-97.

3. Hessel EA, 2nd. A Brief History of Cardiopulmonary Bypass. *Semin Cardiothorac Vasc Anesth* 2014;18(2):87-100.

4. Keltie K, Elneil S, Monga A, et al. Complications following vaginal mesh procedures for stress urinary incontinence: an 8 year study of 92,246 women. *Sci Rep* 2017;7(1):12015.

5. Medicines and Heathcare products Regulatory Agency (MHRA). Medical devices: how to comply with the legal requirements in Great Britain. https://www.gov.uk/guidance/medical-devices-how-to-comply-with-the-legal-requirements#classification-devices (accessed 20th December, 2021).

6. National Institute for Health and Care Excellence (NICE). The National Institute for Health and Care Excellence (NICE) Interventional Procedures Programme. Summary of requirements for the NHS and clinicians. 2017. https://www.nice.org.uk/Media/Default/About/what-we-do/our-programmes/interventional-procedures/Using-new-IPs-requirements-NHS-and-clinicians.pdf (accessed 20th December, 2021).

7. Royal College of Surgeons of England (RCS). Surgical innovation, new techniques and technologies: A guide to good practice. 2019. https://www.rcseng.ac.uk/-/media/files/rcs/standards-and-research/standards-and-policy/good-practice-guides/old-documents/surgical-innovation-new-techniques-and-technologies.pdf (accessed 22nd September, 2021).

8. Hutchison K, Rogers W, Eyers A, et al. Getting Clearer About Surgical Innovation: A New Definition and a New Tool to Support Responsible Practice. *Annals of surgery* 2015;262(6):949-54.

9. Rogers WA, Lotz M, Hutchison K, et al. Identifying surgical innovation: a qualitative study of surgeons' views. *Ann Surg* 2014;259(2):273-8.

10. Blakely B, Selwood A, Rogers WA, et al. Macquarie Surgical Innovation Identification Tool (MSIIT): a study protocol for a usability and pilot test. *BMJ Open* 2016;6(11):e013704.

11. Karpowicz L, Bell E, Racine E. Ethics Oversight Mechanisms for Surgical Innovation: A Systematic and Comparative Review of Arguments. *J Empir Res Hum Res Ethics* 2016;11(2):135-64.

12. Umscheid CA, Margolis DJ, Grossman CE. Key concepts of clinical trials: a narrative review. *Postgrad Med* 2011;123(5):194-204.

13. Medicines and Heathcare products Regulatory Agency (MHRA). Yellow Card Scheme. https://yellowcard.mhra.gov.uk/the-yellow-card-scheme/ (accessed 22nd September, 2021).

14. Barkun JS, Aronson JK, Feldman LS, et al. Evaluation and stages of surgical innovations. *Lancet* 2009;374(9695):1089-96.

For peer review only

15. Skivington K, Matthews L, Simpson SA, et al. A new framework for developing and evaluating complex interventions: update of Medical Research Council guidance. *BMJ* 2021;374:n2061.

16. Hirst A, Philippou Y, Blazeby J, et al. No Surgical Innovation Without Evaluation: Evolution and Further Development of the IDEAL Framework and Recommendations. *Ann Surg* 2019;269(2):211-20.

17. Sedrakyan A, Campbell B, Merino JG, et al. IDEAL-D: a rational framework for evaluating and regulating the use of medical devices. *Bmj* 2016;353:i2372.

18. Khachane A, Philippou Y, Hirst A, et al. Appraising the uptake and use of the IDEAL Framework and Recommendations: A review of the literature. *Int J Surg* 2018;57:84-90.

19. Pennell CP, Hirst AD, Campbell WB, et al. Practical guide to the Idea, Development and Exploration stages of the IDEAL Framework and Recommendations. *Br J Surg* 2016;103(5):607-15.

20. Moher D, Liberati A, Tetzlaff J, et al. Preferred reporting items for systematic reviews and meta-analyses: the PRISMA statement. *PLoS Med* 2009;6(7):e1000097.

21. Shamseer L, Moher D, Clarke M, et al. Preferred reporting items for systematic review and meta-analysis protocols (PRISMA-P) 2015: elaboration and explanation. *Bmj* 2015;350:g7647.

22. McCulloch P, Altman DG, Campbell WB, et al. No surgical innovation without evaluation: the IDEAL recommendations. *Lancet* 2009;374(9695):1105-12.

23. Cousins S, Blencowe NS, Blazeby JM. What is an invasive procedure? A definition to inform study design, evidence synthesis and research tracking. *BMJ Open* 2019;9(7):e028576.

24. The Medical Devices Regulations 2002. https://www.legislation.gov.uk/uksi/2002/618/pdfs/uksi_20020618_en.pdf (accessed 22nd September, 2021).

25. Ouzzani M, Hammady H, Fedorowicz Z, et al. Rayyan-a web and mobile app for systematic reviews. *Syst Rev* 2016;5(1):210.

26. Harris PA, Taylor R, Thielke R, et al. Research electronic data capture (REDCap)--a metadata-driven methodology and workflow process for providing translational research informatics support. *J Biomed Inform* 2009;42(2):377-81.

27. Braun V, Clarke V. Using thematic analysis in psychology. *Qualitative Research in Psychology* 2006;3(2):77-101.

For peer review only

PRISMA-P Checklist

Adapted from [1].

Section and topic	Item No.	Checklist item	Included
**Administrative info.**			
Title:			
Identification	1a	Identify the report as a protocol of a systematic review	✓
Update	1b	If the protocol is for an update of a previous systematic review, identify as such	n/a
Registration	2	If registered, provide the name of the registry (such as PROSPERO) and registration number	n/a
Authors:			
Contact	3a	Provide name, institutional affiliation, e-mail address of all protocol authors; provide physical mailing address of corresponding author	✓
Contributions	3b	Describe contributions of protocol authors and identify the guarantor of the review	✓
Amendments	4	If the protocol represents an amendment of a previously completed or published protocol, identify as such and list changes; otherwise, state plan for documenting important protocol amendments	✓
Support:			
Sources	5a	Indicate sources of financial or other support for the review	✓
Sponsor	5b	Provide name for the review funder and/or sponsor	✓
Role of sponsor or funder	5c	Describe roles of funder(s), sponsor(s), and/or institution(s), if any, in developing the protocol	n/a
**Introduction**			
Rationale	6	Describe the rationale for the review in the context of what is already known	✓
Objectives	7	Provide an explicit statement of the question(s) the review will address with reference to participants, interventions, comparators, and outcomes (PICO)	n/a
**Methods**			
Eligibility criteria	8	Specify the study characteristics (such as PICO, study design, setting, time frame) and report characteristics (such as years considered, language, publication status) to be used as criteria for eligibility for the review	✓

For peer review only

Information sources	9	Describe all intended information sources (such as electronic databases, contact with study authors, trial registers or other grey literature sources) with planned dates of coverage	✓
Search strategy	10	Present draft of search strategy to be used for at least one electronic database, including planned limits, such that it could be repeated	✓
Study records:			
Data management	11a	Describe the mechanism(s) that will be used to manage records and data throughout the review	✓
Selection process	11b	State the process that will be used for selecting studies (such as two independent reviewers) through each phase of the review (that is, screening, eligibility and inclusion in meta-analysis)	✓
Data collection process	11c	Describe planned method of extracting data from reports (such as piloting forms, done independently, in duplicate), any processes for obtaining and confirming data from investigators	✓
Data items	12	List and define all variables for which data will be sought (such as PICO items, funding sources), any pre-planned data assumptions and simplifications	✓
Outcomes and prioritization	13	List and define all outcomes for which data will be sought, including prioritization of main and additional outcomes, with rationale	n/a
Risk of bias in individual studies	14	Describe anticipated methods for assessing risk of bias of individual studies, including whether this will be done at the outcome or study level, or both; state how this information will be used in data synthesis	n/a
Data synthesis	15a	Describe criteria under which study data will be quantitatively synthesised	n/a
	15b	If data are appropriate for quantitative synthesis, describe planned summary measures, methods of handling data and methods of combining data from studies, including any planned exploration of consistency (such as I^2, Kendall's τ)	n/a
	15c	Describe any proposed additional analyses (such as sensitivity or subgroup analyses, meta-regression)	n/a
	15d	If quantitative synthesis is not appropriate, describe the type of summary planned	✓
Meta-bias(es)	16	Specify any planned assessment of meta-bias(es) (such as publication bias across studies, selective reporting within studies)	n/a
Confidence in cumulative evidence	17	Describe how the strength of the body of evidence will be assessed (such as GRADE)	n/a

1. Shamseer L, Moher D, Clarke M, Ghersi D, Liberati A, Petticrew M, et al. Preferred reporting items for systematic review and meta-analysis protocols (PRISMA-P) 2015: elaboration and explanation. BMJ. 2015;350:g7647.

For peer review only

Search Strategy for Ovid MEDLINE

	Searches
1	((stage or phase or framework) adj2 (IDEAL or development or innovat*)).ti,ab.
2	IDEAL-D.ti,ab,kf,hw.
3	1 or 2
4	(invasive or incision or cut or percutaneous or puncture or (natural adj1 orifice)).ti,ab,kf,hw.
5	(endoscop* or colonoscop* or gastroscop* or thoracoscop* or laparoscop* or arthroscop* or bronchoscop*).ti,ab,kf,hw.
6	(catheter or scalpel or surgery or surgical or operat* or interventional).ti,ab,kf,hw.
7	(device* or implant* or prosthe* or robot*).ti,ab,kf,hw.
8	exp Specialties, Surgical/ or exp Endoscopy/ or Radiology, Interventional/ or exp Surgical Procedures, Operative/ or exp "prostheses and implants"/
9	4 or 5 or 6 or 7 or 8
10	(guideline* or guidance or recommendation* or proposal* or regulation* or advice or framework or typology or model or algorithm or evaluat* or determin* or practical method* or method* or concept* or hinder* or hindrance* or difficult* or problem*).ti,ab.
11	exp Guidelines as Topic/ or exp Classification/
12	10 or 11
13	3 and 9 and 12
14	limit 13 to english language

For peer review only

Search Strategy for Embase

	Searches
1.	((stage or phase or framework) adj2 (IDEAL or development or innovat*)).ti,ab.
2.	IDEAL-D.ti,ab.
3.	1 or 2
4.	(invasive or incision or cut or percutaneous or puncture or (natural adj1 orifice)).ti,ab,tw.
5.	(endoscop* or colonoscop* or gastroscop* or thoracoscop* or laparoscop* or arthroscop* or bronchoscop*).ti,ab,tw.
6.	(catheter or scalpel or surgery or surgical or operat* or interventional).ti,ab,tw.
7.	(device* or implant* or prosthe* or robot*).ti,ab,tw.
8.	exp surgery/ or exp endoscopy/ or interventional radiology/ or exp "prostheses and orthoses"/
9.	4 or 5 or 6 or 7 or 8
10.	(guideline* or guidance or recommendation* or proposal* or regulation* or advice or framework or typology or model or algorithm or evaluat* or determin* or practical method* or method* or concept* or hinder* or hindrance* or difficult* or problem*).ti,ab.
11.	exp practice guideline/ or classification/
12.	10 or 11
13.	3 and 9 and 12
14.	limit 13 to english language

For peer review only
